# Making life difficult for Clostridium difficile: augmenting the pathogen’s metabolic model with transcriptomic and codon usage data for better therapeutic target characterization

**DOI:** 10.1186/s12918-017-0395-3

**Published:** 2017-02-16

**Authors:** Sara Saheb Kashaf, Claudio Angione, Pietro Lió

**Affiliations:** 1 Computer Laboratory, University of Cambridge, 15 JJ Thomson Avenue, Cambridge, CB3 0FD UK; 2 Department of Computer Science and Information Systems, Teesside University, Borough road, Middlesbrough, TS1 3BA UK

**Keywords:** Clostridium difficile, Metabolic networks, Metabolic pathways, Metabolic modeling, Genome scale modeling, Flux balance analysis, Sensitivity analysis, Antibiotic resistance

## Abstract

**Background:**

*Clostridium difficile* is a bacterium which can infect various animal species, including humans. Infection with this bacterium is a leading healthcare-associated illness. A better understanding of this organism and the relationship between its genotype and phenotype is essential to the search for an effective treatment. Genome-scale metabolic models contain all known biochemical reactions of a microorganism and can be used to investigate this relationship.

**Results:**

We present *i*cdf834, an updated metabolic network of *C. difficile* that builds on *i*MLTC806cdf and features 1227 reactions, 834 genes, and 807 metabolites. We used this metabolic network to reconstruct the metabolic landscape of this bacterium. The standard metabolic model cannot account for changes in the bacterial metabolism in response to different environmental conditions. To account for this limitation, we also integrated transcriptomic data, which details the gene expression of the bacterium in a wide array of environments. Importantly, to bridge the gap between gene expression levels and protein abundance, we accounted for the synonymous codon usage bias of the bacterium in the model. To our knowledge, this is the first time codon usage has been quantified and integrated into a metabolic model. The metabolic fluxes were defined as a function of protein abundance. To determine potential therapeutic targets using the model, we conducted gene essentiality and metabolic pathway sensitivity analyses and calculated flux control coefficients. We obtained 92.3% accuracy in predicting gene essentiality when compared to experimental data for *C. difficile* R20291 (ribotype 027) homologs. We validated our context-specific metabolic models using sensitivity and robustness analyses and compared model predictions with literature on *C. difficile*. The model predicts interesting facets of the bacterium’s metabolism, such as changes in the bacterium’s growth in response to different environmental conditions.

**Conclusions:**

After an extensive validation process, we used *i*cdf834 to obtain state-of-the-art predictions of therapeutic targets for *C. difficile*. We show how context-specific metabolic models augmented with codon usage information can be a beneficial resource for better understanding *C. difficile* and for identifying novel therapeutic targets. We remark that our approach can be applied to investigate and treat against other pathogens.

**Electronic supplementary material:**

The online version of this article (doi:10.1186/s12918-017-0395-3) contains supplementary material, which is available to authorized users.

## Background

*Clostridium difficile* is a gram-positive, spore-forming, anaerobic bacterium, which infects or colonizes various animal species. Clinical manifestations in humans range from asymptomatic colonization to mild diarrhea, pseudomembranous colitis, and death [1]. Infection by this bacterium is associated not only with significant patient morbidity and mortality, but also with a large economic burden for healthcare systems [2]. The primary risk factor for development of *C. difficile* infection among hospitalized patients is antibiotic use, which promotes toxicogenic *C. difficile* strains to proliferate, produce toxins, and induce disease [3]. Infection by this bacterium is most commonly associated with antibiotics such as clindamycin and amoxicillin [4]. Current recommendations for treatment of *C. difficile* infection (CDI) call for other antibiotics, such as metronidazole for mild infection cases and vancomycin for more severe cases [5]. The emergence of hypervirulent and antibiotic-resistant strains of this bacterium has motivated the search for novel methods of treating CDI. One method involves searching the bacterial central metabolic pathways for drug targets to create the next generation of antibiotics [6].

The quest to better understand this bacterium and identify novel drug targets against it can benefit vastly from a model of the genotype-phenotype relationship of its metabolism. Methods to model the genotype-phenotype relationship range from stochastic kinetic models [7] to statistical Bayesian networks [8, 9]. Kinetic models are limited as extensive experimental data is required to determine the rate laws and kinetic parameters of biochemical reactions. An alternative to kinetic models is metabolic modeling, which has been used to depict a range of cell types without the need for difficult-to-measure kinetic parameters [9]. Metabolic models have been able to predict cellular functions, such as cellular growth capabilities on various substrates, effect of gene knockouts at genome scale [10], and adaptation of bacteria to changes in their environment [11]. Metabolic models require a well-curated genome-scale metabolic network of the cell. Such networks contain all the known metabolic reactions in an organism, along with the genes that encode each enzyme involved in a reaction. The networks are constructed based on genome annotations, biochemical characterizations, and published literature on the target organism. The different scopes of such networks include metabolism, regulation, signaling, and other cellular processes [10].

Despite the success of metabolic modeling in capturing large-scale biochemical networks, the approach is limited as it describes cellular phenotype simply in terms of biochemical reaction rates and is thereby disconnected from other biological processes that impact phenotype. Moreover, metabolic models cannot account for changes in the metabolism of the bacterium in response to different environmental conditions. Recent advances in the omic technologies, such as genomics (genes), transcriptomics (mRNA), and proteomics (proteins), have enabled quantitative monitoring of the abundance of biological molecules at various levels in a high-throughput manner. Integration of transcriptomic data has been shown to be effective in improving metabolic model predictions of cellular behavior in different environmental conditions [12].

Here we present an integrated model of the metabolism of *C.difficile* strain 630. We expanded the network *i*MLTC806cdf [13] with regards to various pathways, such as fatty acid, glycerolipid, and glycerophospholipid metabolism. Fatty acids are not only important components of bacterial cell membranes but they are also important intermediate metabolites in the production of vitamins, lipid A, and quorum sensing molecules [14]. The metabolism of phospholipids is also of interest as these compounds have been found to be closely tied to the growth phase in bacteria such as *Bacillus subtilis* [15, 16].

To bridge the gap between gene expression data and protein abundance, we accounted for the codon usage bias of the bacterium. During translation of a mRNA to a protein, the information contained in the form of nucleotide triplets (codons) in the RNA is decoded to derive the amino acid sequence of the resulting protein. Most amino acids are coded by two to six *synonymous codons*. These codons, which code for the same amino acid, are surprisingly used differentially in protein-encoding sequences [17]. The codon usage has been found to alter the translation time and the abundance of the resulting protein [18, 19]. To our knowledge, this is the first time codon usage has been quantified and incorporated into a genome-scale metabolic reconstruction.

We used the modified network and flux balance analysis [20] to simulate the steady-state metabolism of the bacterium. To understand the behavior of the bacterium in different environments, we integrated gene expression data. We incorporated the codon usage of the bacterium to bridge the gap between gene expression levels and protein abundance in the model. We then validated our metabolic models against the literature on the bacterium. Following this validation process, we used our models to identify potential drug targets. Essential genes have been previously proposed as potential therapeutic targets. [13]. We propose an additional method of predicting therapeutically-relevant genes through metabolic pathway sensitivity analysis and calculation of flux control coefficients. The choice of gene to target can be further refined by eliminating genes with a human homolog to reduce the off-target effects of the selected drug [13].

## Methods

### Construction and validation of the metabolic model

#### icdf834: an expansion of the iMLTC806cdf network

In modifying the *i*MLTC806cdf network [13], we consulted KEGG [21] and incorporated some of the output from the review and curation of the MetaCyc [22] database for *C. difficile*, which was released on March 20, 2015. During curation, we manually considered the directionality and gene-reaction associations of each reaction in the existing network. We also manually expanded the existing network according to the procedure specified by Thiele et al. in [23]. We supported additions to the network with published literature on the bacterium. For example, the fatty acid profile found in *Clostridium difficile* is mostly dominated by C16:0, C16:1, C18:1, and C18:0 [24]. The major phospholipid types in this bacterium are phosphatidylglycerol analogs, with PG(31:2), PG(32:1), PG(33:2), PG(33:1) constituting the majority of these species [24]. Our modified network *i*cdf834 modifies and expands pathways concerning lipid metabolism in the existing network, such as those where compounds and reactions involved had been grouped together. By expanding the metabolism of the bacterium, we can also account for the wide array of fatty acids *C. difficile* can metabolize from its environment. This can provide important insights as many Gram-positive bacteria have been found to be able to incorporate and metabolize extracellular fatty acids [25]. When defining metabolic pathways in the expanded network, we used KEGG pathway identifiers so to remain consistent with the conventions employed in *i*MLTC806cdf [13].

The lipid component of the biomass equation of *i*MLTC806cdf had been obtained from the metabolic network of *Staphylococcus aureus* [26], where lipid compounds had been lumped together. There is a paucity of analyses on the chemical content of *C. difficile*’s biomass. Therefore, upon increasing the granularity of the network, we assumed coefficients from the biomass equation of the *i*Bsu1103 metabolic network developed for *Bacillus subtilis*, where these lumped lipid and teichoic acid species have been replaced by explicit species.

#### Constraint-based reconstruction and modeling approach

One constraint-based method for simulating the metabolic steady-state of a cell is flux-balance analysis (FBA), which can be used to analyze the metabolic network solely on the basis of systemic mass-balance and reaction capacity constraints. FBA simulations have been able to capture microorganism growth, nutritional resource consumption, and waste-product secretion rates of various cell types [27].

The first step of FBA involves representing the metabolic network in the form of a numerical matrix *S* of size (*m*×*n*). This matrix contains the stoichiometric coefficients of each of the *m* metabolites in the *n* different reactions. In the matrix, each row represents one unique metabolite and each column represents one reaction. The stoichiometric matrix helps enforce a mass balance constraint on the system. The mass balance on the cell for *i*=1,…,*m* metabolites and *j*=1,…,*n* reactions constrains the metabolite concentrations *x*_*i*_, as shown in Eq. 1, where *v*_*j*_ is the flux through reaction *j*.

1 $$ \frac{dx_{i}}{dt} = \sum\limits_{j=1}^{n} S_{ij} v_{j}, i=1,\ldots,m.   $$

Under the steady state assumption $\frac {dx_{i}}{dt}=0, \forall i$, the total amount of any compound being produced equals the total amount being consumed:

2 $$ \sum\limits_{j=1}^{n} S_{ij} v_{j}=0, i=1,\ldots,m.   $$

In most metabolic models, there are more reactions than there are compounds [20]. Because there are more unknown variables than equations (*n*>*m*), any *v* that satisfies Eq. 2 is considered to be in the null space of *S*.

FBA can be used to find and determine points within the solution space that are most representative of the biological system using linear programming methods. Studies have revealed that metabolic fluxes in microorganisms are best predicted by maximizing the cellular objectives of growth [27]. To determine the point corresponding to the maximum growth rate within the constrained space, the objective function shown in Eq. 3 was maximized:

3 $$ f(v)=c^{T}v,   $$

where *c* is a vector of weights and indicates how much each reaction flux contributes to the biomass objective function. The maximum growth rate can be achieved by determining the flux distribution *v* that results in maximal biomass flux. Additional constraints can be added through the upper bound $v_{j}^{U}$ and the lower bound $v_{j}^{L}$ for the flux *v*_*j*_. These bounds mandate the minimum and maximum fluxes allowed for a certain reaction and further decrease the space of allowable flux distributions for the relevant system. The mathematical representation of the metabolic reactions, the objective function, and the capacity constraints define a linear system as shown in Eq. 4.

4 $$ \begin{aligned} & {\text{max}} && c^{T}v \\ & \text{subject~to} && Sv=0 \\ &&& v_{j}^{L}\leq v_{j}\leq v_{j}^{U}, \; j = 1, \ldots, n. \end{aligned}   $$

The model fluxes are usually given units of *mmol*/*gDW*·*h*, where *gDW* is the dry weight of cell mass in grams and *h* is the reaction time in hours. The bounds enforce thermodynamic constraints by dictating whether reactions are reversible or irreversible. The lower and upper flux bounds were arbitrarily chosen to be -10 *mmol*/*gDW*·*h* and 10 *mmol*/*gDW*·*h* for reversible reactions. For irreversible reactions, $v_{j}^{L}$ was chosen to be 0 *mmol*/*gDW*·*h* and $v_{j}^{U}$ was set to 10 *mmol*/*gDW*·*h*. For our analysis, we used the COBRA toolbox 2.0 [28] in Matlab (version R2015b, Mathworks, Inc.).

#### Multi-objective optimization in metabolic models

One limitation of using only biomass as the objective is that goals in metabolism are often different and simultaneously competing so the scalar notion of “optimality” does not hold; examples of such trade-offs include maximizing energy production while minimizing protein costs [29]. Moreover, the biomass objective vector is usually perpendicular to one of the surfaces of the solution space of the FBA problem. Consequently, biomass maximizing flux states are usually degenerate; there exist multiple flux distributions that yield the same maximal biomass value [30]. To choose between the various flux distributions, additional criteria must be considered. For these reasons, we modeled metabolism as a multiobjective phenomenon. By modeling the metabolism of bacterium as a multi-objective problem, we address a conflict problem whereby maximizing one objective (eg. biomass) might involve a trade-off in the other objective (eg. intracellular flux); cells are thought to face a trade-off that is described by the set of Pareto-optimal solutions. We used a multi-objective optimization approach to address the *z* objectives, as shown in Eq. 5.

5 $$ \begin{aligned} & {\text{max}} && f(v)=(f_{1}(v), f_{2}(v),\ldots,f_{z}(v))\\ & \text{subject to} && Sv=0 \\ &&& v_{j}^{L}\leq v_{j}\leq v_{j}^{U}, \; j = 1, \ldots, n. \end{aligned}   $$

Note that, without loss of generality, we assumed that all the functions have to be maximized since minimizing a function *f*(*v*) is equivalent to maximizing −*f*(*v*).

Various works have attempted to systematically evaluate the ability of different objectives functions to reliably predict intracellular flux [31, 32]. According to their findings, bacterial metabolism can be better described by the objective of maximization of biomass or ATP production paired with the objective of minimization of intracellular flux [32]. Introducing the minimization of intracellular flux as a secondary objective allows for economic allocation of resources by the bacterium by selecting for metabolic routes that contain the fewest number of steps [33]. Thus, for our analyses we used maximization of biomass, along with minimization of intracellular flux as our objectives.

In a maximization multi-objective problem, a vector that is part of the feasible space is considered to be Pareto-optimal if all other vectors have the same or a lower value for at least one of the objective functions. Therefore, a Pareto-optimal solution is found when there exists no other feasible solution which would increase one objective without decreasing another objective. The set of Pareto-optimal solutions constitutes the Pareto-optimal front [34]. In the absence of additional information, no one Pareto-optimal solution can be said to be better than the other; higher-level information is required to choose one of the solutions [35].

As proposed by Costanza et al. [36], to solve this multi-objective optimization problem one can use bilevel linear programming coupled with evolutionary algorithms, namely stochastic optimization methods that simulate the process of natural evolution. Evolutionary algorithms are well suited to multi-objective problems because they can generate multiple Pareto-optimal solutions after one run and can use recombination to make use of the similarities of solutions [35]. The input to the evolutionary algorithm is a set of arrays, also called *individuals*, representing potential solutions to the problem. These arrays are then ranked based on the values of their objective functions. Potential optimal solutions are generated by retaining the best individuals and by generating new individuals through the use of variation. This process is continued until no further improvements are detected on the Pareto front. The population size and the number of populations used with this algorithm were 140 and 1400, respectively. To solve the linear programs, we used the Gurobi solver (v5.6.3, Gurobi Inc.) [37].

To validate our choice of objectives, we conducted a genetic analysis using multi-objective optimization. In this analysis, binary “knockout” vectors were created, with each containing a 1 in the location of a gene set to be off [36]. This analysis allowed us to determine how the growth of the organism changes in different environments, when genes may be turned on or off.

#### Robustness analysis

A facet of living organisms is their homeostasis, otherwise known as their ability to remain robust to external and internal perturbations within a certain range. External perturbations include changes in temperature or food supply while internal perturbations include spontaneous mutations. The robustness of biological systems is partly due to the presence of parallel metabolic pathways. Robustness represents the insensitivity of a system to changes in system parameters.

Global Robustness (GR) analysis can be used to survey the parameter space to determine the region where the cell exhibits specific features. More specifically, we perturbed the flux bounds of the metabolic model and observed the resulting effects on biomass production. The perturbation function *γ*(*ψ*,*σ*) where *γ* applies noise *σ*, assumed to be Gaussian, to the system *ψ* for the trial *τ*. As proposed in [38], a robust trial is associated with a *ρ* of 1:

6 $$ \rho(\psi,\tau,\phi,\epsilon)= \left\{\begin{array}{ll} 1, &\text{if}~|\phi(\psi)-\phi(\tau)| \leq \epsilon\\ 0, &\text{otherwise} \end{array}\right.  $$

where *ε* is the robustness threshold. The GR was defined as the percentage of trials determined to be robust. We arbitrarily defined *ε* to be 1% of the metric *ϕ*(*ψ*) and we arbitrarily limited the noise to 1%.

#### Incorporating transcriptomic and codon usage data in genome-scale models

**Fig. 1:**
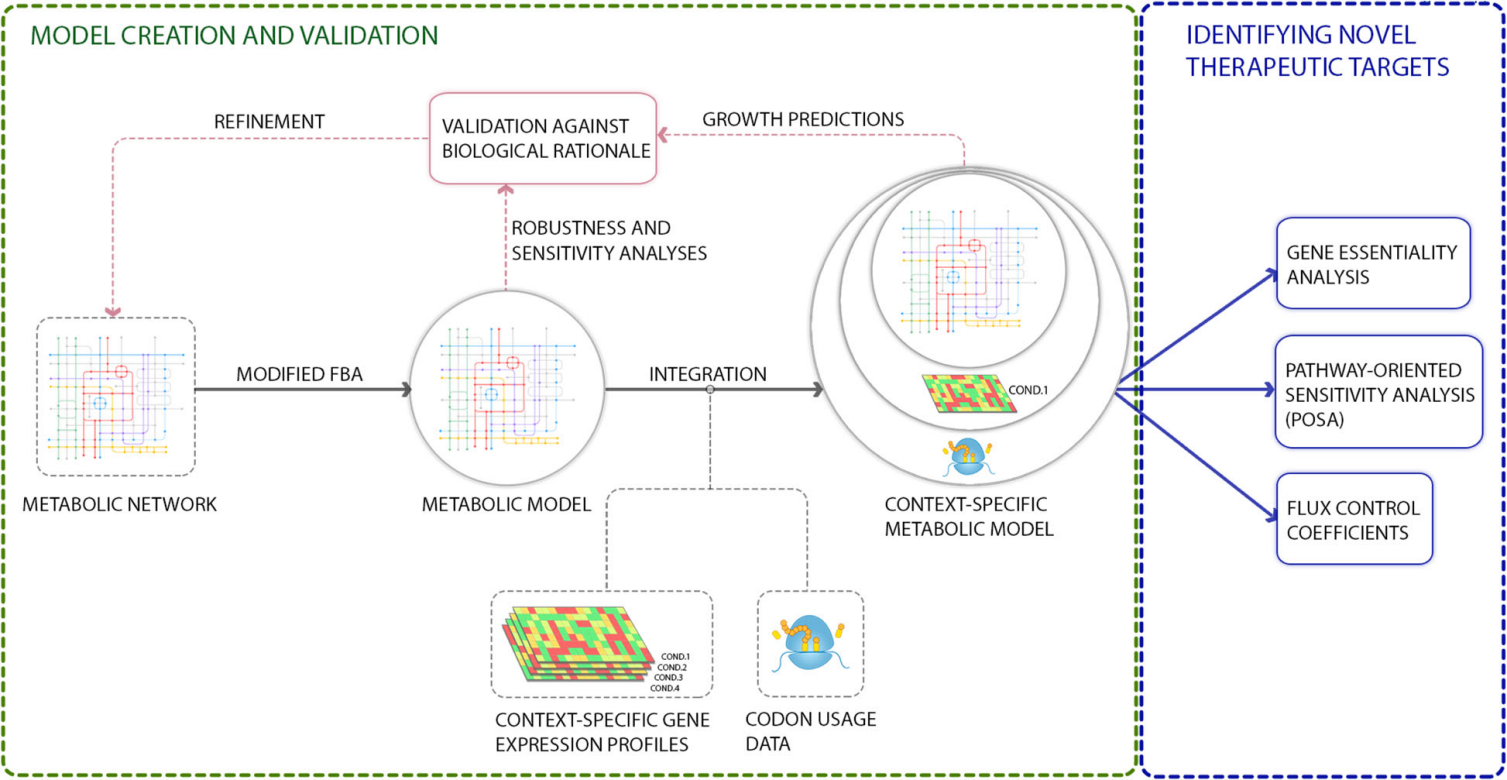
Framework for modeling the metabolism of *C.difficile*. The updated metabolic network of the bacterium was used to create a metabolic model that was assessed using sensitivity and robustness analyses. Integrating gene expression and codon usage data yielded context-specific metabolic models that were evaluated against biological rationale and found fit for clinical applications. The augmented metabolic models were then used to identify potential therapeutic targets using gene essentiality analysis, PoSA, and flux control coefficient calculations

To increase the reliability of the model, gene expression data was added to the FBA framework (Fig. 1). To relate this gene expression data to protein abundance, codon usage bias data was also incorporated. The translation rate of a codon is determined in part by the speed of diffusion of a translationally-competent tRNA to the ribosome. Because tRNAs are differentially abundant in the cell, codons pairing to high-abundance tRNAs are translated faster than those pairing to low-abundance tRNAs. Although synonymous codons produce the same amino acid sequence, they can alter the translation speed and the protein expression levels depending on the abundance of their associated tRNA [39]. Studies have revealed that a large codon bias generally resulted in higher protein expression levels [18, 19]. Therefore, the inclusion of codon bias can help improve the metabolic model predictions by helping link gene expression levels to protein levels.

The codon usage table for *C. difficile* was obtained from the Kazusa Codon Usage Database [40], which lists the frequency of different codons in the genome. The weights for synonymous codons was determined as the ratio between the observed frequency of the codon *k* and the frequency of the most preferred synonymous codon for that amino acid:

7 $$ w_{k}=\frac{f_{k}}{\max(f_{m})}, \text{where } k,m\in\,[\!\text{synonymous~codons}].   $$

We obtained the mRNA sequence associated with the 834 genes of *C. difficile* from UniProt [41]. The counts of different codons were determined for each mRNA sequence. To obtain a measure of the codon bias, we calculated the Codon Adaptation Index (CAI) for each gene. The CAI represents the relative adaptiveness of the codon usage of the relevant gene to the codon usage of highly expressed genes [42]. The CAI ranges from 0 to 1, with a value of 1 indicating high expression and, by correlation, high abundance of the associated protein. The CAI represents the geometric mean of the weights corresponding to the codons in the sequence:

8 $$ CAI=e^{\left[\frac{1}{L}\sum\limits_{l=1}^{L}\ln(w_{k(l)})\right]},  $$

where L is the number of codons in the genes and *w*_*k*(*l*)_ is the weight associated with codon type *k* for *l*th codon along the length L of the gene. Because a large codon bias has been shown to result in higher protein expression levels, the gene expression data *g*_*t*_ for each gene *t* was scaled by *CAI* such that genes with the low codon bias had lower expression $g^{\prime }_{t}$:

9 $$ g_{t}' = g_{t} \cdot (CAI_{t}).  $$

Each of the reactions in the metabolic model depends on a gene set, which is represented through the use of AND/OR operators. In this formulation, if a gene set is composed of two genes and an AND operator, both genes are required to carry out the corresponding reaction. On the other hand, if two genes connected by OR, one gene is sufficient in carrying out the reaction. This formulation can be transformed to derive the gene set expression *GSE*_*j*_ for gene set *j* of reaction *j* from the expression of individual genes *gt*′, which in our case has been scaled by their respective codon usage. When two genes are connected through an AND operator, the gene set expression for reaction *i*, *g*_*i*_, is the minimum of the scaled expression of the individual genes *t* making up the gene set. The gene set expression for two genes connected by an OR operator is the sum of the scaled expression of the individual genes. In each reaction of the model, to map the gene set expression into a specific condition of the model, we used the piecewise muliplicative function *h* and the associated *h*_*j*_ was adopted as a multiplicative factor for the flux bounds [43] :

$$v_{j}^{L} h(GSE_{j})\leq v_{j}\leq v_{j}^{U} h(GSE_{j}), $$

where

10 $$ h(GSE_{j})= \left\{\begin{aligned} &(1+|{log}(GSE_{j})|)^{\frac{GSE_{j}-1}{|GSE_{j}-1|}} \text{if}~GSE_{j} \in \mathbb{R}^{+} \setminus \{1\}\\ &1~\text{if}~GSE_{j}=1\\ \end{aligned}\right.   $$

The function *h* was chosen because at high mRNA abundance, an increase in mRNA abundance has been found to produce a relatively small increase in the protein synthesis rate. On the other hand, at low mRNA abundance, an increase in mRNA abundance has been found to produce a large increase in the protein synthesis rate [44].

Finally, we validated our context-specific metabolic models by incorporating codon usage and differential gene expression data into our model. We then compared trends in our models’ biomass predictions to literature on the bacterium.

### Prediction of therapeutic targets

#### Essential gene analysis

For each gene in the model, essential gene analysis involved removing reactions catalyzed by the gene or by a complex involving that gene and then using FBA [20] to predict growth. Genes were considered essential if following their removal, the predicted maximum growth rate was zero. The *C. difficile* R20291 (ribotype 027), for which gene essentiality data was available for comparison with our in silico results, had been grown on Tryptone-Glucose-Yeast Extract (TGY) broth. To approximate this medium, we used the complex medium defined by Larocque et al. during essential gene analysis of *i*MLTC806cdf [13].

#### Pathway-oriented sensitivity analysis

The growing research attention on metabolic pathways, rather than on specific reactions, is motivated by novel methods that allow for a better understanding of the functionality of complex webs of metabolic reactions. To date, much of the study of metabolic pathways, their crosstalks, and their role in the overall metabotype has been carried out with statistical and model-based approaches [45, 46].

Sensitivity analysis is used to identify model inputs that have a large influence on the model outputs. To find the metabolic pathways that have the largest effect on the outputs of *i*MLTC806cdf and *i*cdf834, we used Pathway-oriented Sensitivity Analysis (PoSA) [36]. PoSA involves genetically manipulating the metabolic model to find the *sensitive* pathways, which make a large impact on model outputs. In other words, we perturbed pathways by mutating the genes that govern their biochemical reactions and analyzed the result on the outputs. In the knockout vector *y*={*b*_1_,*b*_2_,…,*b*_*s*_,…,*b*_*p*_}, *b*_*s*_ represents the perturbations on the genes governing the metabolic pathway *s*, where |*b*_*s*_|=*W*_*s*_ (number of genes partaking in the *s*th pathway). Because the gene knockouts are represented through the use of binary variables, we perform combinatorial perturbations, namely the bits in *b*_*s*_ are switched from 0 to 1 or from 1 to 0; note that if a gene in *b*_*s*_ is set to 1, this gene is knocked-out in the model.

According to [36], the Pathway Elementary Effect (PEE) for the genetic perturbation *b*_*s*_ can be defined as follows:

11 $$ PEE_{s}=\frac{\parallel{F(b_{1},b_{2},\ldots,\tilde{b_{s}},\ldots,b_{p})-F(\tilde{y})}\parallel}{\Delta_{s}},   $$

where $\tilde {b_{s}}$ represents the genetic manipulation of the input *b*_*s*_; $\tilde {y}$ is the mutation carried out on the knockout vector *y*; *F*(*y*) is the vector *v* of fluxes as produced by the model; finally, *Δ*_*s*_ is a scale factor defined as:

12 $$ \Delta_{s}=\frac{1}{W_{s}}\sum_{i=1}^{W_{s}}\tilde{b_{s}}(i), \quad s=1,\ldots,p.  $$

Next, the sensitivity indices *μ* and *σ* are determined by calculating the mean and the standard deviation of the distribution of the PEE for each input. Pathways with a large *μ* have a large influence on the output. A large *σ* indicates an input whose influence highly depends on the value of other inputs. By perturbing the genes through the use of knockouts and comparing the outputs of the model with and without the genetic manipulations, we detected the most sensitive pathways of the metabolic models.

#### Calculation of flux control coefficients

PoSA provides valuable information on sensitive pathways that can be targeted by therapies, but often more specific drug target predictions are desired. To understand how a metabolic pathway is controlled and can be altered, its control structure has to be determined. The *flux control coefficient* [47] is the flux *v*_*ydh*_ through a particular reaction, catalyzed by enzyme *ydh*, of the metabolic pathway with respect to the concentration *x*_*xase*_ of an enzyme xase:

13 $$ \begin{aligned} C^{v_{ydh}}_{x_{xase}}= \frac{\partial {v_{ydh}}}{\partial x_{xase}} \cdot \frac{x_{xase}}{v_{ydh}}= \frac{\partial \ln{v_{ydh}}}{\partial \ln x_{xase}} \end{aligned}   $$

In our calculations, the enzyme concentration was assumed to be equal to the gene expression level adjusted by CAI. When calculating the flux control coefficients, we considered a 1% perturbation in the enzyme concentration *x*_*xase*_. Flux control coefficients provide a quantitative measure of the degree of control an enzyme exerts on a metabolite flux and can quantitatively substitute for the qualitative concept of essential gene [48]. Thus, they can be used to identify steps that should be modified to achieve a successful alteration of the flux in outputs of clinical (e.g. drug therapy) relevance.

### Analysis of cDNA microarrays

We used microarray analysis to determine the combination of genes which were up-regulated or down-regulated in different environmental conditions. We used *Limma* [49], a package in Bioconductor 3.1, for statistical analysis of gene expression. We preprocessed the data through background correction, within-array normalization, and between-array normalization. After normalization, we used filtering to remove probes that did not appear to be expressed in any of the experimental conditions. Next, we used linear models to analyze the microarray data. To conduct statistical analysis and assess differential expression, we used an empirical Bayes method to modulate the standard errors of the log-fold changes. To test for the comparisons of interest, we used an analysis of variance (ANOVA) model.

## Results and discussion

### Expansion and modification of *i*MLT806cdf to *i*cdf834

The genome of *C. difficile* strain 630 is composed of a circular chromosome of 4,290,252 bp coding for 3968 open reading frames (ORFs), along with a plasmid containing 7881 bp coding for 11 ORFs [50]. The modified metabolic network draft contains 21% of the ORFs present in the chromosomal genome of the bacteria with 834 ORFs, a modest improvement upon *i*MLTC806cdf, which contains 806 ORFs, as shown in Table 1. Our expanded metabolic network also consists of 807 metabolites and 1227 reactions. The final version of the network is available as an SBML file and as an Excel file that indicate the reactions, metabolites, genes, and compartments involved in the metabolic network, along with references to literature that support additions or modifications to the existing network. The new network has two additional dead-end metabolites as compared with those found in *i*MLTC806cdf. The Excel and SBML file, along with the the justification for keeping the dead-end metabolites in the model, have been uploaded to http://github.com/ssahebkashaf/Peptoclostridiumdifficile630. The code for all of the analyses employed in our work is also freely available on this repository.

**Table 1:** Comparison of the metabolic network *iMLTC806cdf* published by [13] and the modified and expanded network *i*cdf834

Features		Number	
*Genomic Information of C. difficile*			
	Genome size (bp)	4,290,252	
	Open reading frames	3968	
*Reconstructed models*			
		*iMLTC806cdf*	*icdf834*
	Metabolites	703	807
	Reactions	1091	1227
	Open reading frames	806	834

We repeated analyses previously conducted by Larocque et al. to validate *i*MLTC806cdf [see Additional file 1]. Namely, we compared the ability of *i*cdf834 and *i*MLTC806cdf to identify essential amino acids and metabolizable carbon sources. The removal of amino acids that were not found to be essential or to affect growth, did not affect model-predicted biomass production in both models. Moreover, no biomass was produced in the absence of essential amino acids (cysteine, leucine, isoleucine, proline, tryptophan and valine) [51] in both models. Therefore, similar to the previous network, our network is able to account for the essentiality of various amino acids on the growth of *C.difficile*.

With regards to carbon sources, both models were able to correctly predict a range of carbon sources that are utilized by the bacterium. Moreover, the bacterium was able to generate biomass in the absence of other carbon substrates, such as fructose, mannose, mannitol, and sorbitol. This finding is consistent with literature, which maintains that *C. difficile* is not restricted to metabolizing sugars and can ferment other compounds, even amino acids, to obtain both its carbon and energy [52].

### Validation of metabolic models

#### Genetic analysis using multi-objective optimization

**Fig. 2:**
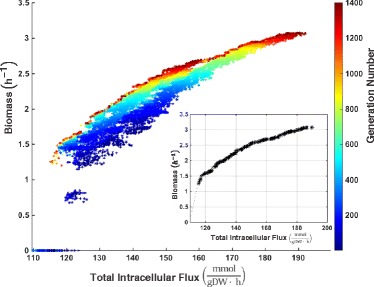
Genetic analysis using multi-objective optimization. Regions of objective space explored by the optimization algorithm for the objectives of maximization of biomass and minimization of total intracellular flux. Solutions are represented by progressively warmer colors depending on the time step of the algorithm in which they had been adaptively generated from the initial point. The Pareto front is shown in *black* in the inset

Our modeling approach is intended to simulate the conflicting objectives faced by the bacterium, where optimal performance in one objective coincides with sub-optimal performance in another objective. We used a knockout parameter space to find the genetic designs that would optimize the two objectives. In Fig. 2, we show the areas of objective space discovered by the genetic algorithm during the genetic analysis from the first generation to generation 1400. The optimization algorithm adaptively moves to regions that maximize biomass while minimizing the total intracellular flux, as evident in the curvature of the plot in Fig. 2. After conducting the genetic analysis, the Pareto front, shown in black in the inset of Fig. 2, was determined. The Pareto front is the set of nondominated solutions that represents the range of phenotypes resulting from different trade-offs between the two objectives. The presence of a Pareto front, as opposed to a singular dominated solution, aligned with our a priori expectations regarding the metabolic plasticity inherent to the bacterium [53]. Our findings, along with previous literature on the choice of objectives, supported our choice of objectives to model *C. difficile*’s metabolism.

#### Robustness analysis

We gauged the robustness of our model by determining the change in the maximal biomass flux in response to different perturbations. Global Robustness (GR) analysis revealed that the biomass production was fully robust to perturbations for a flux bound perturbation (*σ*) and a tolerance (*ε*) of 1% [see Additional file 2]. The GR falls when *σ* is increased or *ε* is decreased. This facet of the bacterium’s metabolism was biologically relevant as bacteria such as *C. difficile* are able to grow despite small fluctuations in their physical environment. Robustness analysis illustrated that the global behavior of our metabolic model matches our expectations from biological rationale and supported the use of our models to predict the behavior of the bacterium in different environments.

#### Changes in C. difficile’s growth in different conditions

We obtained the relevant microarray datasets from the Gene Expression Omnibus (GEO) database [54] under the accession numbers GSE22423 and from the ArrayExpress database [55] under the accession numbers E-GEOD-37442 and E-BUGS-56. Context-specific models for *C. difficile* were generated by incorporating gene expression data obtained for the bacterium in different environmental conditions. To improve the reliability of the model, we also integrated codon usage data. Model predictions of these context-specific models were compared to expectations about the organism’s behavior from literature.

Previous work suggests that sub-MIC concentrations of amoxicillin, metronidazole, and clindamycin slowed growth of toxigenic *C. difficile* as compared with the controls [56]. To test these findings in silico, we incorporated gene expression levels of *C. difficile* in response to sub-MIC levels of different antibiotics into our model. As compared with the *C. difficile* grown on BHI broth, toxigenic strains of *C. difficile* grown on sub-inhibitory concentrations of antibiotics exhibited reductions in their biomass, with those grown on amoxicillin showing the smallest growth (as shown in Table 2). This finding is supported by literature [57] that has shown that in vitro, amoxicillin is effective against *C. difficile*. These findings have lead to speculations that in vivo, this antibiotic is effective aginst vegetative forms of the bacterium but not against *C. difficile* spores [58]. Another potential explanation is that this broad-spectrum antibiotic may impair the intestinal microflora in a way that supports proliferation of *C. difficile*.

**Table 2:** Percent change in model-predicted biomass production (growth) of *C. difficile* in different conditions

Microarray data accession	Condition	% change in
number/database		biomass (*h* ^−1^)
E-GEOD-37442/		
ArrayExpress		
	Heat shock from 30 °C to 43 °C	*↓*24.3%
E-BUGS-56/		
ArrayExpress		
	Sub-MIC level of amoxicillin	*↓*27.4%
	Sub-MIC level of clindamycin	*↓*16.6%
	Sub-MIC level of metronidazole	*↓*2.3%
	BHI broth	*↑*1.0%
GSE22423/GEO		
	Supplementation of 10mM cysteine	*↑*1.1%

The microarray data for each condition was obtained from the GEO or ArrayExpress databases, using the specified accession numbers. The differential gene expression levels obtained from analysis of this microarray data was used to make a metabolic model for each condition. These context-specific metabolic models were used to predict change in biomass production for each condition compared with the control of each microarray dataset

Additionally, the decline in biomass production following heat shock from 30 to 43 °C shown in Table 2 could be due to the general stress response employed by the bacterium. The heat shock response of *C. difficile* has been found to be involve gene clusters homologous to *E. coli* heat-shock operons [59]. The heat shock response in *E. coli* has been found to be associated with a decrease in central carbon metabolism and a decline in cellular growth [60]. Literature on related bacteria is thereby in agreement with the model’s prediction of a significant reduction in growth in *C. difficile* following the heat shock. Additionally, according to the work of Dubois et al., the supplementation of 10mM cysteine to the medium did not affect *C. difficile*’s growth [61]. After integrating the microarray data from their work, we found that our in silico findings agreed with their experimental results.

Validation of the findings of our context-specific metabolic models against the literature on the bacterium showed that metabolic models allow for an enriched view of omic data and may be valuable tools for better understanding the behavior of *C. difficile* in different conditions.

### Prediction of therapeutic targets

#### Gene essentiality analysis

Essential genes have been cited as promising targets for development of new antimicrobials due to their importance for bacterial survival [62]. Using FBA, we performed an in silico gene deletion study to predict potential essential genes that may lead to the identification of new drug targets. This analysis had already been conducted for *i*MLTC806cdf based on a 5% threshold, and gene essentiality results had been compared to genes deemed essential for *B. subtilis*, for which this data had been available [13]. We performed gene essentiality analysis for both *i*MLTC806cdf and *i*cdf834 and validated our results using recently available literature on the essential genes of the *C. difficile* R20291 (ribotype 027) [63]. While *i*MLTC806cdf predicted 48 essential genes and had a 86.5% accuracy in predicting gene essentiality, *i*cdf834 predicted 46 essential genes and had a 92.3% accuracy [see Additional file 3].

#### Pathway-oriented sensitivity analysis and flux control coefficients

**Fig. 3:**
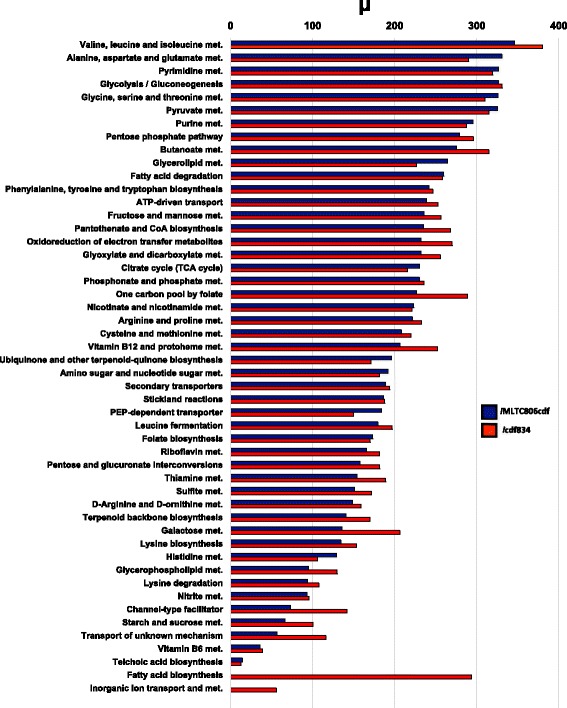
PoSA was used to compare the most sensitive pathways of *i*MLTC806cdf and *i*cdf834. The *i*MLTC806cdf model is composed of 48 metabolic pathways and the *i*cdf834 model is composed of 50 metabolic pathways. Biomass production is most sensitive to pathways with higher calculated *μ*

For our PoSA analysis, we chose the gene expression profile of the bacterium when grown on BHI broth. Each pathway was assessed through random perturbations of its reactions, and the average perturbation *μ* and the standard deviation *σ* were computed as a result. We performed the pathway-based sensitivity analysis and identified sensitive pathways before and after modifying the metabolic model as shown in Fig. 3. The pathway with the largest *μ*, and thereby the greatest control on biomass production or growth in both *i*MLTC806cdf and *i*cdf834 is the valine, leucine, and isoleucine metabolism pathway. These three amino acids are essential to the bacterium and their metabolism was also expected to be essential. The second most sensitive pathway is alanine, aspartate, and glutmate metabolism in *i*MLTC806cdf and glycolysis/gluconeogenesis in *i*cdf834. Additional sensitive pathways in *i*cdf834 include pyrimidine metabolism and pyruvate metabolism. Model findings suggest that therapies against infection may likely be more effective if they target key enzymes in these sensitive pathways.

**Fig. 4:**
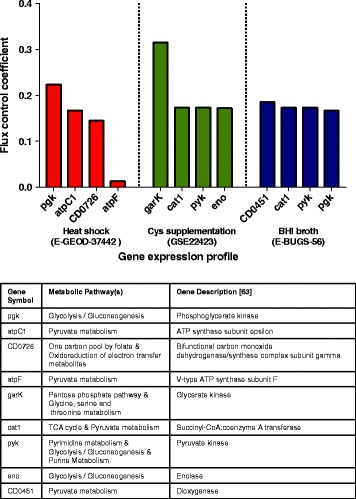
Genes encoding the enzymes with the largest flux control coefficients for biomass production in different conditions (*top*). Table of metabolic pathway(s) hosting the genes and of gene descriptions [64] (*bottom*). A flux control coefficient of 1 implies full control of the metabolite flux by the associated enzyme

To find more specific therapeutic targets, flux control coefficients for enzymes on biomass production in the metabolic model were determined and compared for BHI broth (E-BUGS-56), cysteine supplementation (GSE22423), and heat shock (E-GEOD-37442) gene expression data. The four enzymes with largest flux control coefficients in each condition are shown in Fig. 4, while the complete list of flux control coefficients in different conditions has been uploaded to the public repository. These flux control coefficients were interestingly involved in pathways deemed sensitive during PoSA. These enzymes varied amongst the four conditions, suggesting that access to the in vivo gene expression profile of *C. difficile* can be used to predict better drug targets for patients. Therapies aimed at reducing growth of *C. difficile* should target enzymes with high flux coefficients as, according to our model, their activity is most closely tied to biomass production.

## Conclusion

In this study, we expanded the existing metabolic network for *C. difficile* and used it to create context-specific metabolic models of its metabolism that allow us to understand how the bacterium alters its metabolism depending on its environment. To predict the bacterium’s behavior in different environmental conditions, the model was integrated with transcriptomic and codon usage data to generate reliable and context-specific metabolic flux distributions. We validated the model by conducting robustness and sensitivity analyses. We further assessed its predictive potential by comparing model predictions with published experimental data to gauge the consistency of model findings with the current knowledge of *C. difficile*’s metabolism. Through this literature-based validation, we found that the model is a valuable tool for qualitatively understanding the behavior of the bacterium in different settings. The model can also be used to find potential therapeutic targets by allowing for determination of essential genes and context-specific sensitive pathways and flux control coefficients.

Context-specific metabolic models can allow for a better understanding different medically-relevant conditions (eg. pre-infection, post infection) and can be continuously refined by integrating novel information regarding *C. difficile*’s metabolism. Our model can be used by biomedical researchers to study the bacterium and devise targeted treatments. Our approach can also be scaled-up to simulate the interactions between the gut microbiota and the host using a bottom-up modeling approach. By accounting for the gut microbiota-host interactions, we can construct a whole gut model response to infections and other inflammatory events, paving the path towards more informed and effective treatments.

## Availability and requirements

- **Project name:** *i*cdf834
- **Project home page:** http://github.com/ssahebkashaf/Pep-toclostridiumdifficile630
- **Operating system:** platform independent
- **Programming language:** MATLAB
- **Other requirements:** Gurobi
- **License:** University of Cambridge
- **Any restrictions to use by non-academics:** license needed

## Additional files

Additional file 1 Carbon source usage, carboxylic acids secreted, and essential amino acids in *i*cdf834 as compared with *i*MLTC806cdf and literature. (PDF 41 kb)

Additional file 2 GR of biomass production in *i*cdf834 to internal and external perturbations. (PDF 656 kb)

Additional file 3 Gene essentiality analysis of *i*cdf834 and *i*MLTC806cdf. (PDF 49 kb)

## Abbreviations

CAI: Codon adaptation index
FBA: Flux balance analysis
GR: Global robustness
ORF: Open reading frame
PEE: Pathway elementary effect
PoSA: Pathway-oriented sensitivity analysis
TGY: Tryptone-glucose-yeast extract
