# Supplementary figures and images for: Making life difficult for Clostridium difficile: augmenting the pathogen’s metabolic model with transcriptomic and codon usage data for better therapeutic target characterization
Source: BMC Syst Biol. 2017 Feb 16;11:25. doi: 10.1186/s12918-017-0395-3 (PMC5314682; doi:10.1186/s12918-017-0395-3)

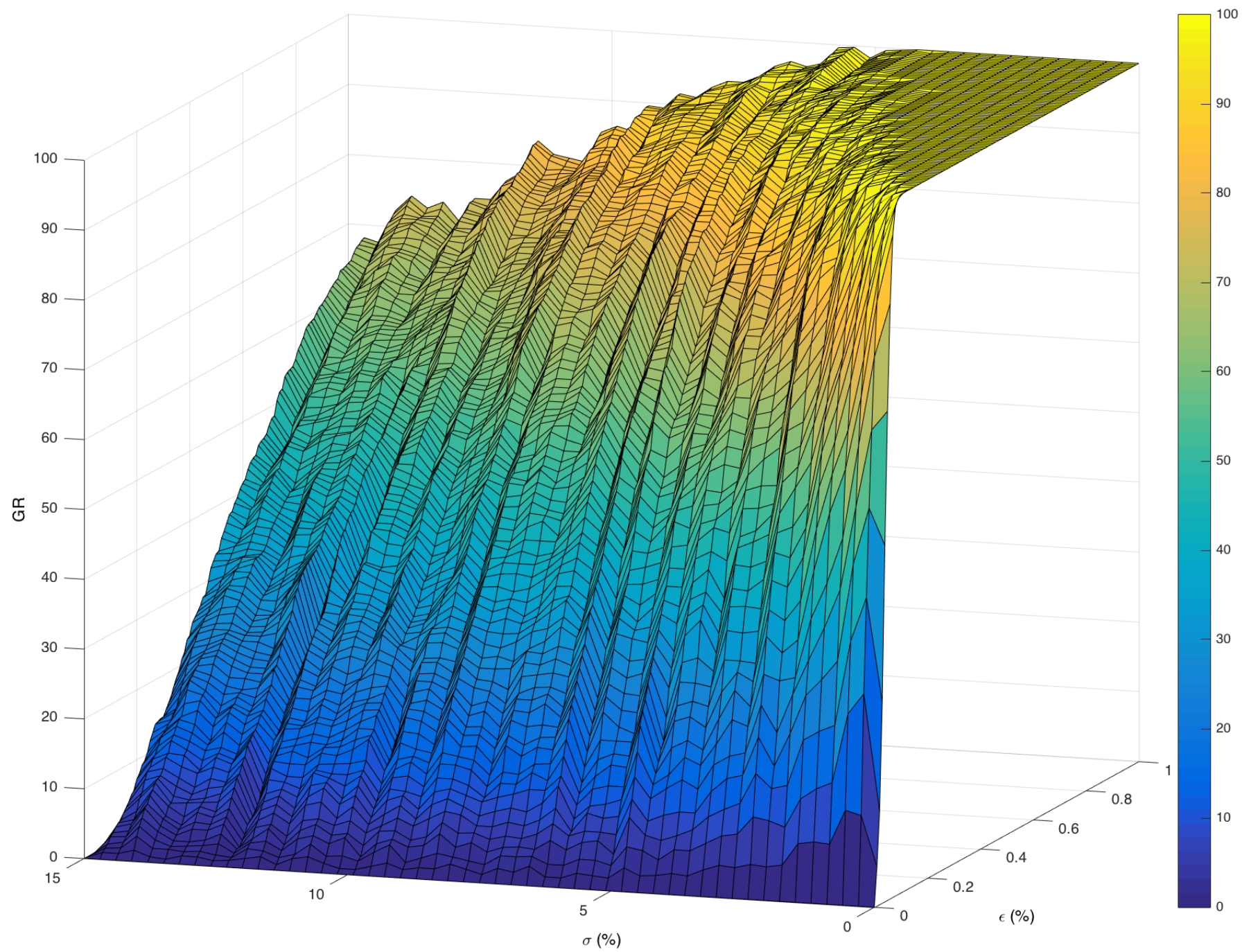

Supplement: Additional file 2 — GR of biomass production in icdf834 to internal and external perturbations. (PDF 656 kb) [file 12918_2017_395_MOESM2_ESM.pdf]
